# Performance Improvement of Atmospheric Continuous-Variable Quantum Key Distribution with Untrusted Source

**DOI:** 10.3390/e23060760

**Published:** 2021-06-16

**Authors:** Qin Liao, Gang Xiao, Shaoliang Peng

**Affiliations:** 1 College of Computer Science and Electronic Engineering, Hunan University, Changsha 410082, China; hnuxg@hnu.edu.cn; 2 Center for Optoelectronic Information Engineering, Central South University, Changsha 410075, China; 3 School of Computer Science, National University of Defense Technology, Changsha 410073, China; 4 Peng Cheng Lab, Shenzhen 518000, China

**Keywords:** continuous-variable quantum key distribution, atmospheric channel, entanglement source, photon subtraction operation.

## Abstract

Atmospheric continuous-variable quantum key distribution (ACVQKD) has been proven to be secure theoretically with the assumption that the signal source is well protected by the sender so that it cannot be compromised. However, this assumption is quite unpractical in realistic quantum communication system. In this work, we investigate a practical situation in which the signal source is no longer protected by the legitimate parts, but is exposed to the untrusted atmospheric channel. We show that the performance of ACVQKD is reduced by removing the assumption, especially when putting the untrusted source at the middle of the channel. To improve the performance of the ACVQKD with the untrusted source, a non-Gaussian operation, called photon subtraction, is subsequently introduced. Numerical analysis shows that the performance of ACVQKD with an untrusted source can be improved by properly adopting the photon subtraction operation. Moreover, a special situation where the untrusted source is located in the middle of the atmospheric channel is also considered. Under direct reconciliation, we find that its performance can be significantly improved when the photon subtraction operation is manipulated by the sender.

## 1. Introduction

Continuous-variable quantum key distribution (CVQKD) [1,2,3,4] is a branch of quantum cryptography, it allows two distant legitimate partners (Alice and Bob) to share an identical secret key over an insecure quantum channel, its security is guaranteed by the laws of quantum mechanics [5,6]. Fiber-based CVQKD has been widely investigated over the past dozen years since the first CVQKD protocol, “GG02” was proposed [7]. According to the research, the theoretical security of fiber-based CVQKD has been proven in both an asymptotic limit [8] and finite-size regime [9]. Recently, the composable security proof for discrete-alphabet fiber-based CVQKD protocols have also been presented [10,11].

With the development of quantum communication technologies, especially after the first quantum satellite “Micius” is launched [12], CVQKD over free-space becomes another research hotspot [13,14]. CVQKD over free-space, especially in an atmospheric channel [15,16,17], can deliver a secret key to any place without the limitation of a fiber link, so that it is more flexible than fiber-based CVQKD. Therefore, investigating CVQKD over an atmospheric channel is beneficial for establishing global quantum communication systems. Recently, the ultimate limits and benchmarks have been established for ACVQKD [18], which provides comprehensive machinery for studying the composable finite-size security of CV-QKD protocols in free-space links (see also [19] for other investigations). However, due to the negative impact of transmission efficiency caused by atmospheric turbulence and the instability of the radiation source [20], the performance of CVQKD over an atmospheric channel is not desirable. The author of [21] suggested a tunable CVQKD scheme for the satellite-to-ground free space optical link using orthogonal frequency division multiplexing technology. Although it can theoretically improve the performance of ACVQKD in terms of the secret key rate, the complicated design is hard to implement with current technologies. The work of [22] proposed another improved approach for ACVQKD, showing that the performance of ACVQKD can be enhanced with the help of a proper non-Gaussian operation. However, all the above-mentioned works are based on an underlying assumption that the signal source cannot be compromised. This is actually quite unpractical in a real quantum system, since legitimate users may also be compromised in a realistic environment, let alone the signal source. Although this issue can be theoretically fixed by applying plug-and-play measurement-device-independent (PP MDI) configuration in which both the measurement device and signal source are integrated to the third untrusted party, Charlie [23], PP MDI-based DM CVQKD actually does not work well in a realistic communication system. This is because the most widely used amplitude modulators, e.g., LiNbO_3_ modulators, are polarization sensitive and features a polarizer, where the light can hardly be transmitted if its orientation is not perfectly aligned in PP configuration. Fortunately, Ref. [24] has proved the theoretical asymptotic security of CVQKD with a signal source in the middle of an insecure fiber link, thereby solving the issue of an untrusted entanglement source [25]. However, although security can be guaranteed, its performance is dramatically reduced.

In this work, we consider a practical configuration of ACVQKD in which a signal source is placed in an insecure atmospheric channel. With this configuration, we consider several situations where the signal source is placed at different positions of the atmospheric channel, and respectively analyze their performance. Unsurprisingly, we find that the performance of ACVQKD is dramatically reduced without the protection of legitimate parts, especially when the signal source is located in the middle of the atmospheric channel. In order to improve the performance of this practical ACVQKD system, photon subtraction [26] is introduced. Photon subtraction, which is a kind of non-Gaussian operation, has been demonstrated theoretically and experimentally to significantly enhance the maximal transmission distance of the CVQKD systems [27,28], and can be easily implemented with current technologies. Numerical simulation shows that the performance of ACVQKD with an untrusted source can be improved by properly adopting the photon subtraction operation. In particular, the performance of ACVQKD with an untrusted source in the middle of a channel can be significantly improved when the photon subtraction operation is applied to the import of the sender.

This paper is organized as follows. In Section 2, we demonstrate the design of the signal source in an untrusted channel, then, an improved ACVQKD by using photon subtraction is proposed. In Section 3, we show the security analysis of the improved ACVQKD. In Section 4, a model of transmission fluctuation in an atmospheric channel is introduced. Subsequently, we analyze the effect of the position of the signal source in an untrusted channel, and give the performance analysis of the ACVQKD with a photon subtraction operation through numerical simulation. Finally, a conclusion is given in Section 5.

## 2. ACVQKD with Untrusted Source and Its Improved Approach

In this section, we investigate a practical situation where the signal source is placed in an untrusted atmospheric channel, and detail the principle of a photon subtraction operation.

### 2.1. ACVQKD with Untrusted Source

In general, the signal source is used for generating a secure key and has to be protected by the trustworthy sender. However, the sender cannot guarantee the security of the signal source in the actual quantum system. In this view, we consider a practical configuration whereby the signal source is moved to the middle (or other location) of the untrusted atmospheric channel. In this configuration, the two-mode squeezed states (Einstein–Podolsky–Rosen (EPR) state) [29,30] serve as the signal source, and the corresponding Gaussian unitary is defined as S(l) = exp$[\frac{l}{2}(\hat{a}\hat{b}-{\hat{a}}^{†}{\hat{b}}^{†})]$, where *l* is the squeezing parameter. The EPR state ${|\Psi \rangle }_{AB}$ is generated by combining two rotated squeezed vacuum states on a balanced beam splitter, and this process can be described as:

(1) $$\gamma_{AB}={\left(Y^{BS}\right)}^{T}\left(\gamma_{A}⊕\gamma_{B}\right)Y^{BS},$$

where $\gamma_{A}$ and $\gamma_{B}$ are the covariance matrices of a squeezed and antisqueezed state, respectively, and $Y^{BS}$ is the operation of the balanced beam splitter. The covariance matrices of $\gamma_{AB}$ is written as:

(2) $$\gamma_{AB}=\left(\begin{array}{cc} VI & \sqrt{V^{2}-1}Z\\ \sqrt{V^{2}-1}Z & VI \end{array}\right),$$

where *I* represents the identity matrix diag(1,1), Z represents matrix $\left[\begin{array}{cc} 1 & 0\\ 0 & -1 \end{array}\right]$, and *V* is the modulation variance and its value can be calculated by $V=\frac{1}{2}\left(V_{S}+V_{A}\right)$. $V_{S}$ is the variance for the squeezed state and $V_{A}$ is for the antisqueezed state.

**Figure 1:**
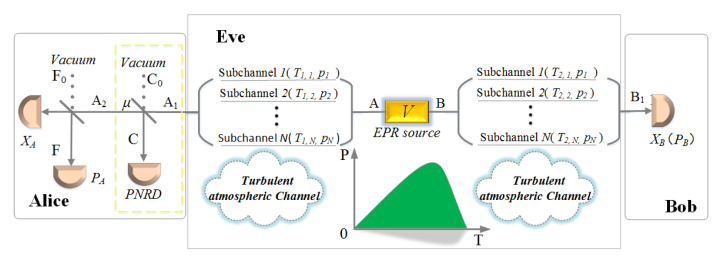
Schematic diagrams of the proposed ACVQKD with an untrusted source. The signal source (EPR) is located in the untrusted atmospheric channel. Each mode of EPR is sent to Alice and Bob respectively. The atmospheric channel is modeled by several subchannels, whose transmittance is fluctuating in time among *T* and occurrence probability *P*. Yellow dotted box presents the module of photon subtraction operation which is located on Alice’s side.

Once an EPR state is prepared, one half of the EPR state is transmitted to Alice through an atmospheric channel, and the other half is also transmitted to Bob through an atmospheric channel. Taking that the transmission of the channel fluctuates randomly and the signal source is moved to the atmospheric channel into consideration, the atmospheric channel is splitted into two halves, each half divided into *N* different subchannels with a constant attenuation. The transmission and possibility of each subchannel between Alice and the EPR source are $T_{1,i}$(0 ≤ $T_{1,i}$ ≤ 1) and $p_{i}$, and those between the EPR source and Bob are $T_{2,i}$(0 ≤ $T_{2,i}$≤ 1) and $p_{i}$. The relationship of subchannels is $\sum_{i=1}^{N}p_{i}=1$, and schematic diagrams of the configuration is depicted in Figure 1.

The initial EPR state is represented in Equation (2). Therefore, the covariance matrix of the transmitted state in the certain *i*th subchannel can be described as:

(3) $$\gamma_{A_{1}B_{1},i}=\left(\begin{array}{cccc} V_{A_{1}X,i} & 0 & C_{X,i} & 0\\ 0 & V_{A_{1}P,i} & 0 & C_{P,i}\\ C_{X,i} & 0 & V_{B_{1}X,i} & 0\\ 0 & C_{P,i} & 0 & V_{B_{1}P,i} \end{array}\right),$$

where

(4) $$\begin{array}{cc} \; & V_{A_{1}X,i}=V_{A_{1}P,i}=\frac{1}{2}T_{1,i}\left(V_{A}+V_{S}\right)+1-T_{1,i}+T_{1,i}\epsilon ,\\ \; & V_{B_{1}X,i}=V_{B_{1}P,i}=\frac{1}{2}T_{2,i}\left(V_{A}+V_{S}\right)+1-T_{2,i}+T_{2,i}\epsilon ,\\ \; & C_{X,i}=-C_{P,i}=\frac{1}{2}\sqrt{T_{1,i}T_{2,i}}\left(V_{A}-V_{S}\right). \end{array}$$

This convex mixture is a post-selected state, which is then used in security analysis. According to the Wigner function, it and its components can be represented by:

(5) $$\begin{array}{cc} \; & W\left(X,P\right)=\sum_{i}^{N}p_{i}W_{i}\left(X,P\right),\\ \; & W_{i}\left(X,P\right)=\frac{\exp(-\frac{1}{2}X_{i}^{T}V_{X,i}^{-1}X_{i}-\frac{1}{2}P_{i}^{T}V_{P,i}^{-1}P_{i})}{4\pi^{2}\sqrt{detV_{X,i}detV_{P,i}}}, \end{array}$$

where $X=\left(x_{A_{1}},x_{B_{1}}\right),P=\left(p_{A_{1}},p_{B_{1}}\right)$, and the matrices $V_{X,i}$ and $V_{P,i}$ are given by:

(6) $$V_{X,i}=\left(\begin{array}{cc} V_{A_{1}X,i} & C_{X,i}\\ C_{X,i} & V_{B_{1}X,i} \end{array}\right),V_{P,i}=\left(\begin{array}{cc} V_{A_{1}P,i} & C_{P,i}\\ C_{P,i} & V_{B_{1}P,i} \end{array}\right).$$

From Equation (5), the second moments of the quadrature can be derived through integration:

(7) $$\begin{array}{cc} \langle \hat{X},\hat{P}\rangle & =\int W(x_{A_{1}},x_{B_{1}},p_{A_{1}},p_{B_{1}})xpdx\\ \; & =\sum_{i}^{N}p_{i}\int W_{i}\left(x_{A_{1}},x_{B_{1}},p_{A_{1}},p_{B_{1}}\right)xpdx\\ \; & =\sum_{i}^{N}p_{i}{\langle \hat{X},\hat{P}\rangle }_{i}. \end{array}$$

Since the mean value of the initial vacuum-squeezed state is null, the variances are directly governed by the second moments. According to Equation (7), the second moments of Equation (3) are linear combinations of the transmission factors $\sqrt{T_{1,i}}$, $\sqrt{T_{2,i}}$, $T_{1,i}$, and $T_{2,i}$. Therefore, we can use their expected values $\langle T_{1}\rangle$, $\langle T_{2}\rangle$, $\langle \sqrt{T_{1}}\rangle$, and $\langle \sqrt{T_{2}}\rangle$ to replace $T_{1,i}$, $T_{2,i}$, $\sqrt{T_{1,i}}$, and $\sqrt{T_{2,i}}$, respectively. The expected values can be calculated by:

(8) $$\langle \sqrt{T_{m}}\rangle =\sum_{i}^{N}p_{i}\sqrt{T_{m,i}}\;\left(m\in \left\{1,2\right\}\right)$$

and

(9) $$\langle T_{m}\rangle =\sum_{i}^{N}p_{i}T_{m,i}.$$

### 2.2. Photon Subtraction Operation

As the signal source is no longer protected by the sender, the entanglement of EPR may be affected by an untrusted environment, resulting in a performance degeneration of the ACVQKD system. Fortunately, previous research has shown that the entanglement of EPR can be enhanced by a proper photon subtraction operation. To solve the above-mentioned issue, we therefore introduce a photon subtraction operation to improve the performance of ACVQKD with an untrusted source. According to the process ofa photon subtraction operation, the imporved Wigner function can be represented by:

(10) $$W\left(x_{A_{2}},p_{A_{2}}\right)=\frac{1}{P_{S}\left(k\right)}\sum_{i=1}^{N}p_{i}P_{S,i}\left(k\right)W_{i}\left(x_{A_{2}},p_{A_{2}}\right),$$

where $P_{S}\left(k\right)$ is the total success probability of subtracting *k* photons. $P_{S}\left(k\right)=\sum_{i}^{N}p_{i}P_{S,i}\left(k\right)$, $P_{S,i}\left(k\right)$ is the success probability of subtracting *k* photons in the *i*th subchannel, which can be calculated by:

(11) $$\begin{array}{cc} P_{S,i}\left(k\right) & =\left(1-\theta^{2}\right)\sum_{n=k}^{\infty }\theta^{2n}C_{n}^{k}\mu^{n-k}{\left(1-\mu \right)}^{k}\\ \; & =\left(1-\theta^{2}\right){\left(\frac{1-\mu }{\mu }\right)}^{k}\sum_{n=k}^{\infty }{\left(\theta^{2}\mu \right)}^{n}C_{n}^{k}\\ \; & =\frac{1-\theta^{2}}{1-\mu \theta^{2}}{\left[\frac{\theta^{2}\left(1-\mu \right)}{1-\mu \theta^{2}}\right]}^{k}, \end{array}$$

where $C_{n}^{k}$ is the combinatorial number, μ is the transmittance of the balanced splitter (BS) in the photon subtraction operation, and the value of θ can be calculated by $V_{A_{1}X,i}=\left(1+\theta^{2}\right)/\left(1-\theta^{2}\right)$. The relationship between $P_{S,i}\left(k\right)$ and μ is shown in Figure 8.

For the photon subtraction operation on each subchannel, Alice uses a BS with transmittance μ to split $A_{1,i}$ and the vacuum state $C_{0}$ into modes $A_{2,i}$ and *C*, after that, we get a mixed tripartite state $\rho_{A_{2}CB_{1},i}$, expressed as:

(12) $$\rho_{A_{2}CB_{1},i}=U_{BS}{[|\psi \rangle }_{A_{1}B_{1},i}{\langle \psi |}_{A_{1}B_{1},i}⊗|0\rangle \langle 0|]U_{BS}^{†},$$

where ${|\psi \rangle }_{A_{1}B_{1},i}$ is the output state in the *i*th subchannel before the photon-subtraction operation. Alice then uses the positive operator-valued measure (POVM)$\left\{{\hat{\Pi }}_{0},{\hat{\Pi }}_{1}\right\}$ [31] to measure the state *C* in the photon number-resolving detector (PNRD), with states $A_{2,i}$ and $B_{1,i}$ kept only when the POVM element ${\hat{\Pi }}_{1}$ clicks. Therefore, the covariance matrix $\gamma_{A_{2}B_{1},i}$ of the state $\rho_{A_{2}B_{1},i}$ is obtained by:

(13) $$\gamma_{A_{2}B_{1},i}=\left(\begin{array}{cccc} V_{A_{2}X,i} & 0 & C_{X,i}^{{}^{\prime }} & 0\\ 0 & V_{A_{2}P,i} & 0 & C_{P,i}^{{}^{\prime }}\\ C_{X,i}^{{}^{\prime }} & 0 & V_{B_{1}X,i}^{{}^{\prime }} & 0\\ 0 & C_{P,i}^{{}^{\prime }} & 0 & V_{B_{1}P,i}^{{}^{\prime }} \end{array}\right),$$

where

(14) $$\begin{array}{cc} \; & V_{A_{2}X,i}=V_{A_{2}P,i}=\frac{\sqrt{\mu }\theta \left(k+1\right)}{1-\mu \theta^{2}},\\ \; & V_{B_{1}X,i}^{{}^{\prime }}=V_{B_{1}P,i}^{{}^{\prime }}=\frac{\mu \theta^{2}+2k+1}{1-\mu \theta^{2}},\\ \; & C_{X,i}^{{}^{\prime }}=-C_{P,i}^{{}^{\prime }}=\frac{\mu \theta^{2}\left(2k+1\right)+1}{1-\mu \theta^{2}}. \end{array}$$

The detailed calculation can be found in [32].

After the analysis of the photon subtraction operation on each subchannel, the covariance matrix of the improved system $A_{2}B_{1}$ can be described as:

(15) $$\gamma_{A_{2}B_{1}}=\left(\begin{array}{cccc} V_{A_{2}X} & 0 & C_{X}^{{}^{\prime }} & 0\\ 0 & V_{A_{2}P} & 0 & C_{P}^{{}^{\prime }}\\ C_{X}^{{}^{\prime }} & 0 & V_{B_{1}X}^{{}^{\prime }} & 0\\ 0 & C_{P}^{{}^{\prime }} & 0 & V_{B_{1}P}^{{}^{\prime }} \end{array}\right),$$

where the elements are given by:

(16) $$\begin{array}{cc} \; & V_{A_{2}X}=V_{A_{2}P}=\frac{1}{P_{S}\left(k\right)}\sum_{i=1}^{N}p_{i}P_{S,i}\left(k\right)V_{A_{2}X,i},\\ \; & V_{B_{1}X}^{{}^{\prime }}=V_{B_{1}P}^{{}^{\prime }}=\frac{1}{P_{S}\left(k\right)}\sum_{i=1}^{N}p_{i}P_{S,i}\left(k\right)V_{B_{1}X,i}^{{}^{\prime }},\\ \; & C_{X}^{{}^{\prime }}=-C_{P}^{{}^{\prime }}=\frac{1}{P_{S}\left(k\right)}\sum_{i=1}^{N}p_{i}P_{S,i}\left(k\right)C_{X,i}^{{}^{\prime }}. \end{array}$$

In addition, the module of photon subtraction (the yellow dotted box in Figure 1) can also be deployed on Bob’s side, so that we can obtain a different covariance matrix about the mixed state $\rho_{A_{2}B_{1}}$ due to the symmetry of the EPR source in the atmospheric channel CVQKD.

## 3. Calculation of the Secret Key Rate

In this section, we present the calculation of secret key rates of ACVQKD with an untrusted source under direct reconciliation [24]. Since the signal source is moved to the channel, there are two links in the whole ACVQKD system. In order to reduce the difficulty of analysis, we assume that the situation that occurs on these two links is identical, and the security analysis we considered is based on the case that the fading channel is only affected by constant attenuation, which means that the transmitted state still retains the Gaussian property. However, the attenuation of the quantum state is randomly fluctuating due to the environmental factors. Therefore, we use *N* subchannels to describe such a fluctuating channel on each link. In addition, after the photon subtraction operation on the Gaussian mixed state $\rho_{A_{1}B_{1}}$, the derived state $\rho_{A_{2}B_{1}}$ is no longer to hold the Gaussian property. Fortunately, the secret key rate of $\rho_{A_{2}B_{1}}$ is more than that of the Gaussian mixed state. Based on the above analysis, the calculation formula of secret key rate can be given by:

(17) $$K=P_{S}\left(k\right)\left[\beta I\left(A_{2}:B_{1}\right)-\chi_{E}\right],$$

where β is the reconciliation efficiency, $I(A_{2}:B_{1})$ is the Shannon mutual information between Alice and Bob, and $\chi_{E}$ is the Holevo bound of the mutual information between Alice and Eve. Subsequently, the atmospheric channel can be characterized by the covariance matrix $\gamma_{A_{1}B_{1},i}$. Since the first moments of the squeezed state in both quadratures are zero, $\gamma_{A_{1}B_{1},i}$ directly depends on the second moments. Therefore, the covariance matrix $\gamma_{A_{1}B_{1}}$ of the transmitted state is calculated by:

(18) $$\gamma_{A_{1}B_{1}}=\left(\begin{array}{cc} \langle T_{1}\rangle \left(V+H_{1}\right)I & \langle \sqrt{T_{1}}\rangle \langle \sqrt{T_{2}}\rangle \sqrt{\left(V^{2}-1\right)}Z\\ \langle \sqrt{T_{1}}\rangle \langle \sqrt{T_{2}}\rangle \sqrt{\left(V^{2}-1\right)}Z & \langle T_{2}\rangle \left(V+H_{2}\right)I, \end{array}\right)$$

where $H_{m}=\left(1-\langle T_{m}\rangle \right)/\langle T_{m}\rangle +\epsilon$. The fluctuating channel can be regarded as a nonfading channel with transmittance $T_{f}={\langle \sqrt{T_{1}}\rangle }^{2}$ and the channel-added noise can be estimated by $\epsilon_{f}=\left(\langle T_{1}\rangle -{\langle \sqrt{T_{1}}\rangle }^{2}\right)\left(V+\epsilon -1\right)$.

After the photon subtraction operation has been performed, the covariance matrix $\gamma_{A_{1}B_{2}}$ should be considered, as described in Equations (15) and (16). According to the standard form of the EPR state, some elements can be written as:

(19) $$\begin{array}{cc} \; & a=V_{A_{2}X},\\ \; & b=V_{B_{1}X}^{{}^{\prime }},\\ \; & c=C_{X}^{{}^{\prime }}. \end{array}$$

Then the expression of the mutual information between Alice and Bob is represented by:

(20) $$\begin{array}{cc} I(A_{2}:B_{1}) & =\frac{1}{2}\log\frac{V_{A}+1}{V_{A|B}+1}\\ \; & =\frac{1}{2}\log\frac{a+1}{a+1-c^{2}/b}. \end{array}$$

As for the calculation of the Holevo quantity $\chi_{E}$, assuming that Eve purifies the quantum system $A_{2}B_{1}$, so $\chi_{E}=S\left(E\right)-S\left(E|A_{2}\right)$, the calculation can be simplified as:

(21) $$\chi_{E}=\sum_{i=1}^{2}G\left(\frac{\zeta_{i}-1}{2}\right)-\sum_{i=3}^{4}G\left(\frac{\zeta_{i}-1}{2}\right),$$

where

(22) G(x)=(x+1)log(x+1)−xlogx.

$S\left(E\right)=S\left(A_{2}B_{1}\right)$ is the function of the symplectic eigenvalues $\zeta_{1,2}$ of $\gamma_{A_{2}B_{1}}^{G}$, $\zeta_{1,2}^{2}$ can be calculated by:

(23) $$\zeta_{1,2}^{2}=\frac{1}{2}\left[\Delta \pm \sqrt{\Delta^{2}-4D^{2}}\right],$$

with the denotations:

(24) $$\begin{array}{cc} \; & \Delta^{2}=a^{2}+b^{2}-2c^{2},\\ \; & D^{2}=ab-c^{2}. \end{array}$$

$S\left(E|A_{2}\right)=S\left(B_{1}F|A_{2}\right)$ is the function of the symplectic eigenvalues $\zeta_{3,4}$, where *F* is Alice’s auxiliary mode used for the heterodyne detection. The symplectic eigenvalues $\zeta_{3,4}^{2}$ can be calculated by:

(25) $$\zeta_{3,4}^{2}=\frac{1}{2}\left[A_{m}\pm \sqrt{A_{m}^{2}-4B_{m}}\right],$$

with $A_{m}=\left(a+bD+\Delta \right)/\left(a+1\right)$ and $B_{m}=\left(D\left(b+D\right)/\left(a+1\right)\right)$. Based on the above formula, the Holevo information bound $\chi_{E}$ for homodyne detection is estimated.

## 4. Performance Analysis and Disscussion

### 4.1. Fluctuating Loss Due to the Atmospheric Environment

**Figure 2:**
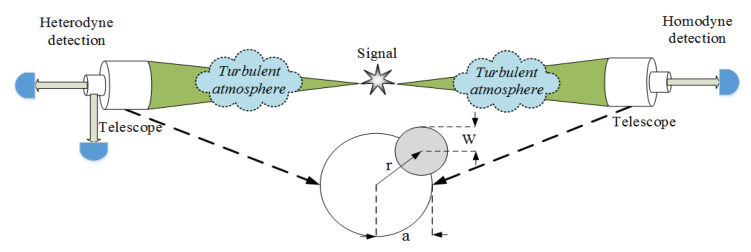
The signal is generated by the entanglement source placed in the atmospheric channel, and transmitted to Alice and Bob through the turbulent atmospheric channel, and finally detected with the help of a telescope. Note that the variation of the beam deflection distance *r* is the main reason for the fluctuation of transmittance.

The atmospheric channel is different from the fiber channel, thus it is necessary to consider the impact of transmission fluctuations caused by the atmospheric environment. In fact, the transmission fluctuation of the atmospheric channel is related to several factors, such as beam wandering, spreading, deformation, and scintillation [18,33,34], and these factors are usually caused by atmospheric turbulence and the instability of the radiation source. To simplify the analysis, here we only consider an important phenomenon of beam wandering [35,36], and the model of Figure 2 well describes the case of beam wandering.

**Figure 3:**
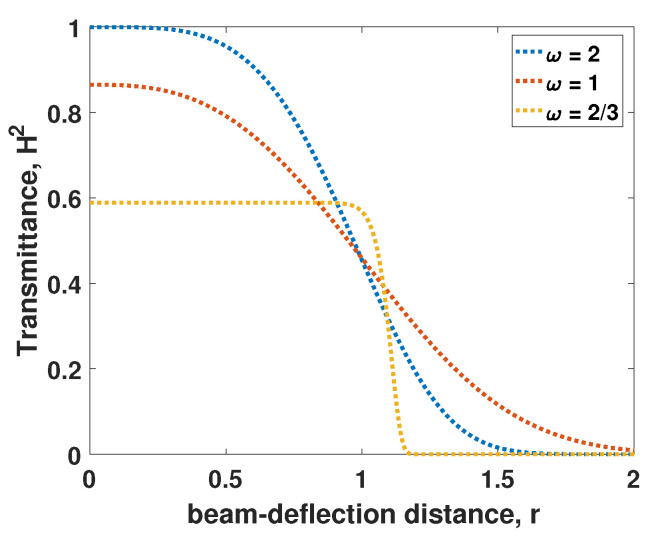
The relationship between transmittance squared and the beam deflection distance *r* for different values of the ratio ω.

Under this circumstance, the expression of the transmission efficiency is approximately given by:

(26) $$H^{2}=H_{0}^{2}\exp\left[-{\left(\frac{r}{R}\right)}^{\lambda }\right],$$

where *r* and *R* respectively represent the beam-deflection distance and scale parameter, and λ represents the shape. Parameter $H_{0}$ represents the maximal transmission coefficient, and has a relationship with the beam-spot radius *W*, calculated by:

(27) $$H_{0}^{2}=1-\exp\left(-2\frac{h^{2}}{W^{2}}\right),$$

where *h* represents the aperture radius. When the beam-deflection distance *r* equals zero, it is obvious that the transmission efficiency is determined by the ratio ω = h/W and cut at $H_{0}$ from Equations (26) and (27). For simplicity, we take some fixed values for ω, and then get the distribution of $H^{2}$ with respect to the beam-deflection distance *r*, as shown in Figure 3. We can find that the transmittance decreases with beam-deflection distance and has a lower maximum with a larger ratio ω.

According to Ref. [37], the beam-deflection distance *r* is decided by the Rice distribution [38], which is described as P(H) by the variance $\delta^{2}$ and the aperture center distance *d*. In addition, P(H) will reduce to a log-negative Weibull distribution when the beam fluctuates around the center of the aperture (*d* = 0), and this distribution is written as:

(28) $$P\left(H\right)=\frac{2R^{2}}{\lambda H\delta^{2}}{\left(2\ln\frac{H_{0}}{H}\right)}^{\frac{2}{\lambda }-1}\exp\left[-\frac{R^{2}{\left(2\ln\frac{H_{0}}{H}\right)}^{\frac{2}{\lambda }}}{2\delta^{2}}\right]$$

except for *H*∈ [0, $H_{0}$] and P(H) = 0.

Based on Ref. [39], the mean value of the transmission efficiency $\langle H^{2}\rangle =\int_{0}^{H_{0}}H^{2}P\left(H\right)dH$ and the mean of the square root of transmission efficiency $\langle H\rangle =\int_{0}^{H_{0}}HP\left(H\right)dH$. Note that the values of $\langle T_{m}\rangle$ and $\langle \sqrt{T_{m}}\rangle$ in Equation (18) can be calculated by $\langle H^{2}\rangle$ and 〈H〉, respectively. In addition, according to the calculation of the Pirandola–Laurenza–Ottaviani–Banchi (PLOB) bound [40], one can bound the secret key capacity of the fading channel by means of the following free-space formula [18]:

(29) $$K\leq \int_{0}^{H_{0}}H^{2}P\left(H\right)\Phi \left(H^{2}\right)\frac{R^{2}}{\lambda H^{2}\delta^{2}}{\left(\ln\frac{H_{0}}{H^{2}}\right)}^{\frac{2}{\lambda }-1}\exp\left[-\frac{R^{2}{\left(\ln\frac{H_{0}}{H^{2}}\right)}^{\frac{2}{\lambda }}}{2\delta^{2}}\right]dH,$$

where $\Phi \left(H\right)=-\log\left(1-H^{2}\right)$.

### 4.2. Parameter Optimization

**Figure 4:**
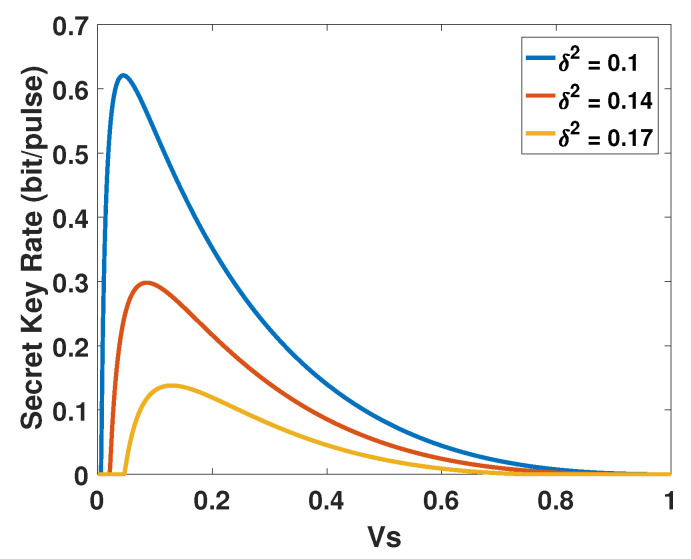
The effect of $V_{S}$ on the secret key rate of CVQKD in the beam wandering case.

After analyzing the characteristics of the atmospheric channel, the parameters of ACVQKD system should be considered. The variance of the signal source is an important parameter since the effective fluctuation-induced noise is variance dependent, and its optimization is crucial for extending the secure distance in free-space link. In Figure 3, we obtain the relationship between beam-deflection distance *r* and transmission efficiency $H^{2}$, and find that transmission efficiency $H^{2}$ has the best performance with ratio ω=2. Therefore, we fix some parameters, such as ω=2, and then study the relationship between the signal source variance and secret key rate. In Figure 4, we show the relationship between $V_{S}$ and the secret key rate of the different parameters $\delta^{2}$ in case of the original protocol (without photon subtraction operation). We find that the secret key rate curves will rise sharply, no matter the value of parameter $\delta^{2}$, the final infinity approaches 0. Which means that the applicable values of squeezing are sensitive to the fluctuating transmittance. The simulation shows that each of the curves is around Vs = 1/12, leading to the maximized secret key rate. All simulation parameters needed for simulation are presented in Table 1.

### 4.3. The Impact of Signal Source Location and Photon Subtraction Operation on ACVQKD

**Figure 5:**
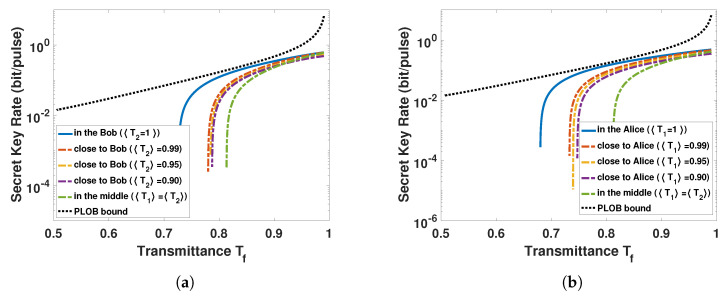
The secret key rate of the ACVQKD as a function of channel equivalent transmittance. (**a**) The untrusted source is located close to Bob, (**b**) the untrusted source is located close to Alice. The blue solid lines in (**a**,**b**) represent the signal source is generated by Bob whose security is trustworthy ($\langle T_{2}\rangle$ = 1) and Alice whose security is trustworthy ($\langle T_{1}\rangle$ = 1), respectively. All dash-dotted lines indicate that the signal source is placed close to Alice or Bob, the green dash-dotted lines represent the signal source is placed in the middle of atmospheric channel, and the black dotted lines represent the maximum secret key capacity on the beam wandering case.

Since the signal source is moved to the atmospheric channel, the location of the signal source has an important effect on the performance of ACVQKD. We first consider the performance of ACVQKD with an untrusted signal source in three different situations: (1) The untrusted source is located close to Alice, (2) the untrusted source is located close to Bob, and (3) the untrusted source is located at the middle of channel. Figure 5 shows that the performance of ACVQKD (with a trusted source) outperforms the above three situations. This result matches with our expectation, as the untrusted source introduces extra noise. In addition, with the decrease of the channel transmittance $\langle T_{2}\rangle$, the secret key rate is too difficult to exist in a lower channel transmittance, and it achieves the worst when the signal source is placed in the middle of the atmospheric channel. The same trend occurs when the signal source is placed close to Alice. The reason is that the system suffers more losses from the atmospheric environment due to the entanglement source being moved from the trusted legitimate party to the atmospheric channel, and the noise increases with the distance between the signal source and the legitimate party. Therefore, the photon subtraction operation is introduced to improve the performance of ACVQKD with the untrusted source.

**Figure 6:**
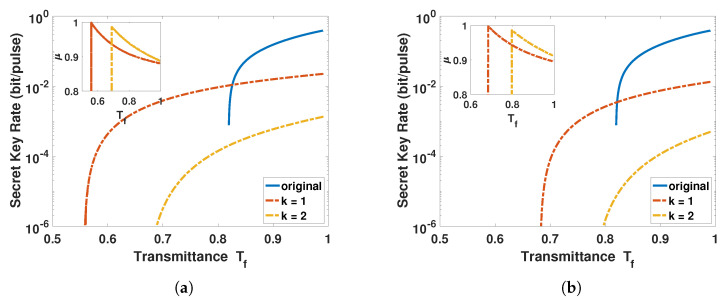
The secret key rate of the ACVQKD as a function of equivalent transmittance $T_{f}$ where the signal source is placed in middle of the atmospheric channel. (**a**) The photon-subtraction operation is deployed to Alice’s side and (**b**) the photon-subtraction operation is deployed to Bob’s side. The blue solid lines represent the case of the original ACVQKD without applying the photon-subtraction operation, the red dash-dotted lines represent the one-photon subtraction, and the yellow dash-dotted lines represent the two-photon subtraction.

**Figure 7:**
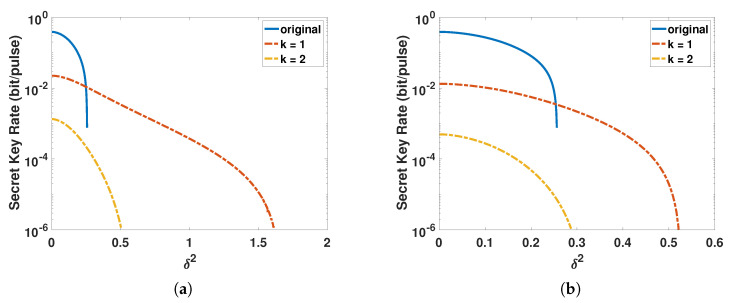
The secret key rate of the ACVQKD as a function of the variance of channel fluctuation $\delta^{2}$ where the signal source is placed at the middle of the atmospheric channel. (**a**) The photon-subtraction operation is deployed to Alice’s side and (**b**) the photon-subtraction operation is deployed to Bob’s side. The blue solid lines represent the case of the original ACVQKD without photon subtraction, the reddash-dotted lines represent the one-photon subtraction, and the yellow dash-dotted lines represent the two-photon subtraction.

In order to reduce the duplication and unnecessary work, we only focus on the worst case that the signal source is placed in the middle of the atmospheric channel. Note that the module of photon subtraction, the yellow box of Figure 1, can also be deployed to Bob’s side. Therefore, there are two different ACVQKD configurations. In Figure 6 and Figure 7, we optimize the *μ* of the BS in the photon subtraction operation to achieve the maximum key rate at different channel transmittance, and the insets represent the optimal transmittance *μ* of BS as a function of channel transmittance.

In Figure 6, when transmittance $T_{f}$ > 0.815, the original ACVQKD outperforms ACVQKD with the photon subtraction operation, no matter whether the photon subtraction operation is deployed on Alice’s side or Bob’s side. The reason is that the photon subtraction operation cannot improve the performance of ACVQKD on the low-loss channel. Furthermore, since the success probability of the photon subtraction operation is relatively low, it further reduces the performance of ACVQKD. When transmittance $T_{f}$ < 0.815, the secret key rate of original ACVQKD is reduced to 0, but there is still a relatively high secret key rate for the ACVQKD with the photon subtraction operation, which illustrates that the photon subtraction operation can tolerate lower channel transmittance with a relatively high secret key rate, so that it is more applicable in the real atmospheric environment. In addition, the performance of ACVQKD with one-photon subtraction operation is better than that of ACVQKD with two-photon subtraction operation, as shown in Figure 6a, it means that the success probability will be reduced and more noises will be introduced due to the risen number of subtracted photons, resulting in a worse performance. In Figure 6b, we can find that the effect of photon subtraction on Bob’s side is not as good as that on Alice’s side. The reason is that the module of the photon subtraction can be regarded as a trusted noise, which has a certain impact on the security of the key. When the photon subtraction operation is deployed to Bob’s side, this trusted noise not only reduces the mutual information between Alice and Bob, but also increases the upper bound of Holevo, when compared to the photon subtraction operation deployed to Alice’s side.

On the other hand, as the aperture center distance *d* = 0, the factors of the fluctuating channel depend on the variance $\delta^{2}$. Figure 7 shows the relationship between the secret key rate and variance $\delta^{2}$, and the photon subtraction operation cannot enhance the ability of channel to resist fluctuation when $\delta^{2}$ < 0.256, no matter whether the photon subtraction operation is deployed on Alice’s side or Bob’s side. However, when 0.256 < $\delta^{2}$, the photon subtraction operation can really enhance the ability of the ACVQKD system to resist channel fluctuation.

**Figure 8:**
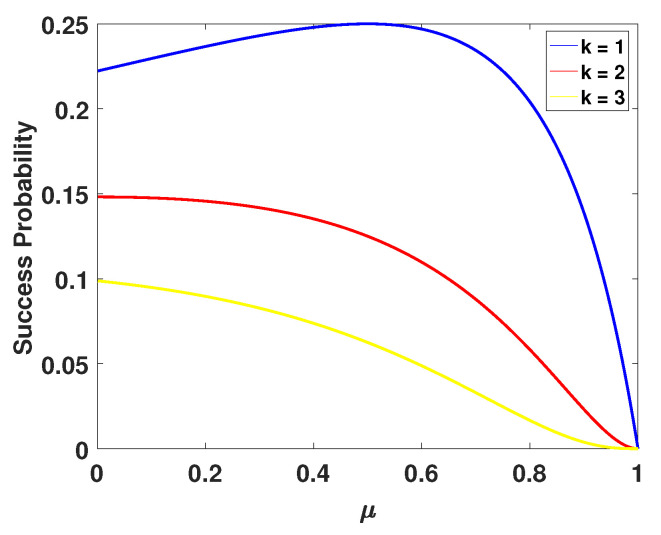
The success probability of subtracting *k* photons with different transmittances μ of BS. The curve surfaces from top to bottom represent one-photon subtraction, two-photon subtraction, three-photon subtraction respectively.

The other significant factor should be noted is the success probability of the photon subtraction operation. According to Equation (17), we find that it plays an important role in the calculation of secret key rates. Figure 8 shows that the success probability of the photon subtraction operation is reduced as the risen numbers of the subtracted photon, and the maximum success probability is less than 0.25. This means that a considerable amount of information will be discarded when performing the photon subtraction operation. In future, the performance and application of our scheme will be improved effectively if the success probability of the photon subtraction operation can be increased.

**Figure 9:**
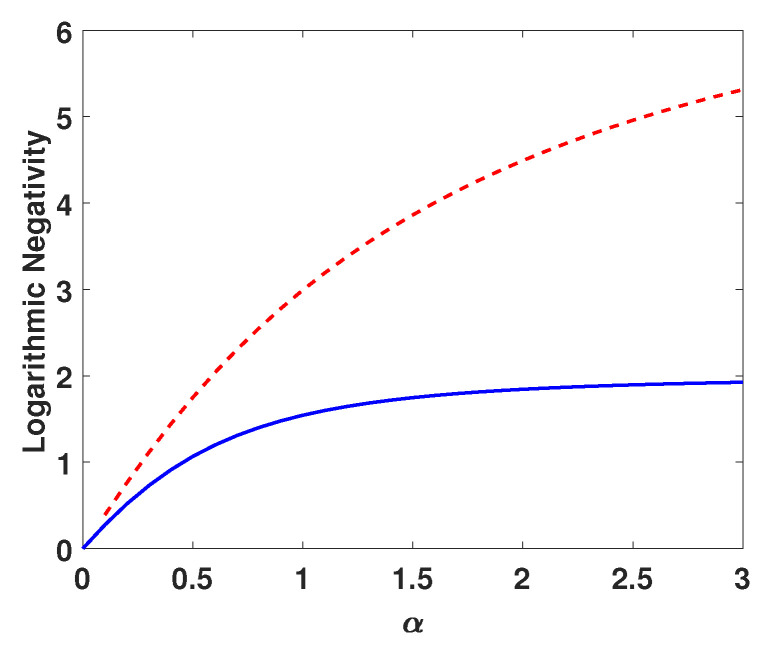
Comparison of the logarithmic negativity for state ${|\Psi \rangle }_{AB}$ [solid (blue) curve] and $|\Psi^{\left(1\right)}\rangle_{AB}$ for the photon subtraction operation [dashed (red) curve] as the function of α with μ = 0.95.

Now, we explore the reason as to why the introduction of the photon subtraction operation can improve the performance of the ACVQKD scheme. We consider the entanglement evolution of the two output modes of the two-mode squeezed state under the photon subtraction operation, and we use the logarithmic negativity as entanglement measures, which has known is an upper bound on the distillable entanglement. The logarithmic negativities of EPR state ${|\Psi \rangle }_{AB}$ and photon subtracted non-Gaussian state $|\Psi^{\left(1\right)}\rangle_{AB}$ can be calculated as:

(30) $$E{(|\Psi \rangle }_{AB})=-\log_{2}\left(1+\alpha^{2}\right)-2\log_{2}\left(\sqrt{1+\alpha^{2}-\alpha }\right)$$

and

(31) $$E{(|\Psi^{\left(1\right)}\rangle }_{AB})=\frac{1+\alpha^{2}\left(1-\mu \right)}{\alpha \sqrt{\mu (1+\alpha^{2})}}\sum_{n=1}^{\infty }\sqrt{n}{\left(\frac{\alpha \sqrt{\mu }}{\sqrt{1+\alpha^{2}}}\right)}^{n},$$

where α=sinhl. As shown in Figure 9, the photon-subtracted non-Gaussian state $|\Psi^{\left(1\right)}\rangle_{AB}$ has a larger amount of entanglement than the EPR state ${|\Psi \rangle }_{AB}$, and the gap extends with μ. In this sense, the photon subtraction operation has improved the correlation between the two modes of bipartite states. However, the photon subtraction operation can effectively improve the performance of ACVQKD based on if the channel attenuation factor being constant, if the attenuation factor is statistically fluctuating, its effect may be unsatisfactory.

## 5. Conclusions

In this paper, we considered a practical situation of ACVQKD where the signal source is not protected by the legitimate parts, but is placed in an untrusted atmospheric channel. By removing the assumption that the signal source cannot be compromised, we found that the performance of ACVQKD with the untrusted source was dramatically degenerated especially when the signal source was placed at the middle of the atmospheric channel. Subsequently, a photon-subtraction operation, which is one of the non-Gaussian operations, was introduced. We showed that proper photon-subtraction operation could enhance the performance of ACVQKD with an untrusted source, especially when it was deployed to Alice’s side. We thus provided a theoretical ground for applying ACVQKD to a realistic environment. 

## Figures and Tables

**Table 1:** Parameter settings for simulation of the secret key rate (all the variances and noises are in shot noise units).

$V_{S}$	β	*h*	*W*	ϵ
1/12	0.9	1	0.5	0.01
